# Thrombotic Events Associated to Aspirin Therapy

**DOI:** 10.1155/2012/247363

**Published:** 2011-11-24

**Authors:** Christian Doutremepuich, Omar Aguejouf, Vanessa Desplat, Dominique Duprat, Francisco X. Eizayaga

**Affiliations:** Laboratoire d'Hématologie, Université Bordeaux 2, 146 Rue Léo Saignat, 33076 Bordeaux Cedex, France

## Abstract

Acetyl salicylic acid (ASA) is widely used in clinical practice. Previous studies done in rats showed unexpected thrombotic potencies of this drug used at ultra-low doses. This review is the first report in which the effects of a wide range of ASA concentration on a microvessel model of laser-induced thrombus formation and Induced Hemorrhagic Time in animals were largely studied.

## 1. Introduction

The antithrombotic effectiveness of aspirin is related to its inhibition of the cyclooxygenase (COX) enzyme that metabolizes arachidonic acid to a variety of prostanoids, including thromboxane A_2_ [1]. Platelet-derived cyclooxygenase-1 (COX-1) generates thromboxane A_2_, a potent vasoconstrictor and platelet agonist. The effect of aspirin on platelet COX-1 is irreversible, thus providing for once-daily low-dose effectiveness. With the inhibition of platelet COX-1 activity, there is a decrease in platelet aggregation, leading to a reduced thromboembolic potential and a commensurate prolonged bleeding time. Thus, it is not surprising that the major risks associated with aspirin relate to bleeding complications.

Several animal studies showed that aspirin used at strong concentrations (100 mg/kg) prevents thromboembolic complications, and this effect is associated with the important hemorrhagic side effects; these effects were also observed in clinical studies [2, 3].

Recently published surveys have warned against the increased risk of aspirin discontinuation, which includes acute coronary problems, stent stenosis, acute myocardial infarction, ischemic stroke, and lower limb ischemia [4–12].

To explain these events we hypothesized a rebound effect. After one dose of 100 mg/kg of aspirin, rats were studied with a Laser-Induced Thrombosis (LIT) model every 2 days for a total period of 16 consecutive days. In the groups studied at days 8 and 10, increased thromboembolic complications were found, corroborating the epidemiological evidence [13]. Similar results were published recently, showing that discontinuation of aspirin, but not thienopyridines, significantly increased the risk of stent stenosis around 7 days after withdrawal [14]. Increased stent thrombosis was described by Eisenberg as being caused by variability in platelet response or a rebound effect. This increased risk of stent thrombosis added to increased acute myocardial infarction and stroke risk makes this effect especially dangerous. This prothrombotic mechanism is not well understood. In another study, we hypothesized that this effect could be due to the residual effects of aspirin. The use of extremely low concentrations of aspirin proved to be as prothrombotic as the effect observed several days after one high dose of aspirin [2]. 

In an attempt to explain these prothrombotic effects, we studied LIT with selective inhibitors of COX 1 (SC-560) and 2 (NS-398). The effect of low concentration aspirin was more marked after COX 1 inhibition and was blunted after COX 2 selective inhibition. Moreover, COX 2 selective inhibition and aspirin at ultra low concentrations had similar prothrombotic effects on the rat [15], suggesting that this effect was possibly related to COX 2 inhibition [16]. To confirm these results, we hypothesized the inhibition of COX 2 by ultra-low concentrations of aspirin and designed an experiment using 72 normal male mice, 72 genetically modified male homozygote mice without COX 1 (COX 1 −/−), and 72 genetically modified male homozygote mice without COX 2 (COX 2 −/−), where we studied induced hemorrhage time (IHT) and LIT to evaluate primary hemostasis.

## 2. Effects of COX Selective Inhibition in Association with Aspirin in Rats

### 2.1. Material and Methods

#### 2.1.1. Animals

Male Wistar rats (200–250 g) purchased from Delpre Breeding Center (St. Doulchard, France) were housed separately and acclimatized before use under conditions of controlled temperature (25 ± 2°C) and illumination (12 h light/dark cycle). They were fed with standard rat chow and water *ad libitum*. Animals received care in compliance with the European Convention of Animal Care.

#### 2.1.2. Induced Hemorrhagic Time

An experimental model of Induced Hemorrhagic Time (IHT) was performed 10 minutes before laser-induced thrombosis. The rat tail was immersed in water for 5 minutes at 37°C and sectioned 6 mm from the extremity. The IHT measured corresponded to the time between tail sectioning and the end of bleeding, expressed in seconds.

#### 2.1.3. Thrombus Induction

Animals were anesthetized with 200 mg/kg of thiopental sodium (Pentothal, Abbott Laboratories, France), and a median laparotomy was performed. The intestinal loop was placed on the microscope table and vascular lesions were induced by an argon laser (Stabilite 2016, Spectra Physics, France). The wavelength used was 514 nm and the energy was adjusted to 120 mW. The laser beam was applied for 1/15 sec. The dynamic course of thrombus formation was continuously monitored and recorded by placing the laser beam coaxially into the inverted light beam path of the microscope (Axiovert, Zeiss, France). Microscopic images were recorded through a digital camera (Basler, Vision Technologies, DX L107, color camera CCD) coupled to a computer and a Dell monitor. A schematic view of the apparatus used has been previously described [17]. Arterioles between 15 and 25 *μ*m diameter were used. The parameters assessed were the number of emboli removed by blood flow and the duration of embolization (time between first and last emboli occurring during a 10-minute observation period).

#### 2.1.4. Drugs Tested

The amounts of 1 mg/mL and 100 mg/mL are obtained by dilution of a solution of Acetylsalicylate (Aspegic, Sanofi-synthelabo, France). Aspirin dilutions were prepared as follows: 1 g of pure, finely powdered aspirin was suspended in 99 mL of alcohol (70°). After being vigorously shaken, 1 mL of this dilution was then mixed with 99 mL of distilled water and vigorously shaken (dilution 1). The last process was repeated until obtaining desired dilutions. After being vigorously shaken, 1 mL of this dilution was then mixed with 99 mL of distilled water and vigorously shaken. The last process was repeated until obtaining desired dilutions: Dil 5, Dil 9, and Dil 15. Sterilized water (placebo) or aspirin were subcutaneously administered at a final volume of 1 mL/kg. The groups were treated with placebo or aspirin in 100 mg/kg, 1 mg/kg or dilutions 5, 9, or 15. 

Selective inhibitors of COX 1, SC-560, and of COX 2, NS-398 were purchased from Cayman Chemical, (Ann Arbor Michigan, USA). They were administered *per os* at doses of 2.5, 5.0, 7.5, or 10 mg/kg body weight, suspended in 0.5% carboxymethylcellulose (CMC) at a final volume of 1 mL/kg. The CMC solution was used as placebo.

#### 2.1.5. Protocol

Two hundred Wistar rats were randomly divided into 20 groups (*n* = 10 rats/group). SC-560 or NS-398 (2.5; 5; 7.5; 10 mg/kg) or association of both inhibitors (10 mg/kg) was administered intragastrically 90 min before the laser procedure. One ultra-low dose of aspirin 1 mL/kg b.w. was injected subcutaneously, 60 min before the laser procedure. Separated groups were injected with placebo.

#### 2.1.6. Statistical Analysis

Data are expressed as Mean ± Standard Error (SEM) and were compared using Student's parametric *t*-test or a one-way ANOVA test followed by Dunnett's or Bonferroni's multiple comparison test when appropriate (*P* < 0.05 was considered significant). GraphPad Prism version 4.0 (GraphPad Software, San Diego, Calif, USA) was used for statistical analysis.

### 2.2. Results

#### 2.2.1. Effects of Products Tested on Induced Hemorrhagic Time

The administration of ULDA reduced the IHT when compared to the control group (*P* < 0.002). The administration of a COX-1-specific inhibitor (SC-560) at different doses significantly and dose dependently increased the IHT (*P* < 0.01). This hemorrhagic side effect of the COX-1-specific inhibitor was neutralized by the administration of ULDA.

The administration of a COX-2-specific inhibitor (NS-398) with or without ULDA did not significantly modify the IHT when compared to placebo. ULDA shortened IHT in the respective group when compared to groups with 2.5, 5, and 7.5 mg/kg of NS 398 and placebo (*P* < 0.01). The group with 10 mg/kg of Ns 398 had an effect similar to ULDA. 

The combined administration of SC-560 and NS-398 resulted in a prolonged IHT (*P* < 0.01), an effect that was normalized by ULDA injection. The result of all the three treatments was a mild nonsignificant modification of the effect of ULDA.

#### 2.2.2. Effects of Products Tested on Laser-Induced Thrombosis

Compared to the control, the administration of ULDA significantly increased the number of emboli (*P* < 0.001) and the duration of embolization (*P* < 0.01). On the other hand, the administration of a COX-1-specific inhibitor (SC-560) significantly decreased the number of emboli (*P* < 0.05) and the duration of embolization (*P* < 0.05) compared to the control. However, this antithrombotic effect of this COX-1-specific inhibitor was neutralized by the administration of ULDA.

In contrast, the administration of a COX-2-specific inhibitor (NS-398) compared to the control group significantly increased the number of emboli at three doses studied (*P* < 0.05) and affected the duration of embolization (*P* < 0.05) at the two highest doses used. ULDA administration did not enhance the prothrombotic effect of this COX-2-specific inhibitor.

The administration of both inhibitors significantly decreased the number of emboli (*P* < 0.01) and the duration of embolization (*P* < 0.01) when compared to placebo and increased the IHT (*P* < 0.01). These effects were normalized by the injection of ULDA. 

The mechanism of this effect is not clearly understood. As COX inhibition is the main mechanism of the effect of aspirin, COX inhibitors were used to try to understand the action of ULDA. COX 1 is a constitutive enzyme responsible for the production of TXA_2_ in platelets. For the above-explained reasons, COX 1 inhibition exerts an antithrombotic influence. COX 2, though inducible, has been claimed to be constitutive in many tissues, including the vascular endothelium. The chronic selective inhibition of COX 2 has been blamed for being prothrombotic and causing serious vascular complications such as myocardial infarction and stroke [18].

ULDA exerted a prothrombotic effect in the control groups, significantly shortened IHT and significantly increased the number of emboli and the duration of embolization. COX 1 selective inhibition had a clear antithrombotic effect expressed as a decreased number of emboli, decreased duration of embolization, and a prolonged IHT. This action is probably expressing TXA_2_ production inhibition in the platelet. Despite this antithrombotic effect, injection of ULDA induced a clear, significant trend to normalization of these values. COX 1 inhibition with sc-560 showed a dose-effect curve by prolonging IHT with each COX 1 inhibitor dose increment. Almost each value at different doses of inhibitor was decreased after ULDA administration, the difference being more pronounced at the higher doses. The data suggest that COX 1 inhibition and ULDA have opposite effects and that COX 1 inhibition does not reduce the effect of ULDA. 

The inhibition of COX 2 with NS-398 produced the opposite effects to laser-induced thrombus formation, since there was a clear prothrombotic activity as shown by an increased number of emboli and longer duration of embolization. Shear stress is surely increased in the laser-induced thrombosis method, as the lesion decreases the diameter of the vessel, thereby increasing velocity and shear stress. Although a slight non-significant increase in IHT was observed with the lowest doses of NS 398, the highest doses led to values close to placebo values. The COX 2 inhibitor and ULDA had similar effects in the laser-induced thrombosis study and in IHT with the highest dose of COX 2 inhibitor. There was no change in the response to ULDA after any of the four different doses of NS-398 with regard to number of emboli, duration of embolization, or IHT, suggesting a common pathway of effect. When the combination of both COX inhibitors was used in the same rat, the result was not substantially different from the results obtained only with COX 1 inhibition. The present study suggests that the selective inhibition of COX 1 or combined inhibition of COX 1 and 2 have clear antithrombotic effects, as observed by a prolonged IHT and the increased laser-induced thrombosis. However, COX 2 selective inhibition and ULDA showed a powerful prothrombotic action in the laser-induced thrombosis study. As demonstrated in the above-mentioned surveys, the thromboembolic complications were observed several days after aspirin withdrawal. A similar delay of 8 to 10 days was observed for a prothrombotic effect in the laser-induced study after a single administration of an antithrombotic dose of aspirin (100 mg/kg b.w.) in the rat [13]. The possibility of a late inhibition of endothelial COX 2 could be the link between the prothrombotic effect of aspirin and the effect of ULDA. Laser-induced thrombosis seems to be a very sensitive method for analyzing the *in vivo* interaction of platelets and endothelium and the production of thrombi in the microcirculation. 

In conclusion, ULDA administered alone demonstrates prothrombotic activities increases the number of emboli and the duration of embolization. COX-1-specific inhibition has antithrombotic and hemorrhagic effects in rats. These effects were modified by ULDA administration. Added, COX-2-specific inhibition has prothrombotic activities that were not enhanced by the administration of ULDA. These results suggest that the prothrombotic effects of ULDA may occur via a COX-2 pathway rather than via a COX-1 route. This would explain the reported thromboembolic complications observed several days after aspirin withdrawal, a phenomenon usually interpreted as a rebound effect. However, this study suggests that this thromboembolic complication is more likely a direct effect of ULDA via COX-2 inhibition.

## 3. Effect of Aspirin in COX 1 −/− or COX 2 −/− Knockout Mice

### 3.1. Material and Methods

#### 3.1.1. Animals

Normal mice from centre d'élevage (Depre Saint Doulchard, France) and the male homozygous COX 1 −/− and COX 2 −/− mice purchased from Taconic Farms Inc. (Hudson City Centre, NY, USA) were housed separately under conditions of controlled temperature and illumination. Knockout animal is an animal into which one specifically introduces, by homologous recombination, a modification into the coding structure of gene or his regulating elements in order to inhibit or to modify the operation of gene in the studied organization.

They were fed with standard mouse chow and water *ad libitum*. Animals received care in compliance with the European Convention of Animal Care.

#### 3.1.2. Induced Hemorrhagic Time (IHT)

IHT was performed 10 minutes before thrombosis induction by laser. The tail of the mouse was immersed in water for 5 minutes at 37°C and sectioned 6 mm from the extremity and is expressed as the time between the tail section and the end of bleeding, expressed in seconds.

#### 3.1.3. Thrombus Induction

Animals were anaesthetized with 200 mg/kg of Ketamine (Panpharma, France). After laparotomy, the intestinal loop was placed on the microscope and vascular lesions were induced by Argon laser (Stabilite 2016, Spectra Physics, France). Two parameters were assessed: the number of emboli (NE) removed from the thrombus by blood flow after an injure produced by the laser shot and the duration of embolisation (DE), expressed in minutes.

#### 3.1.4. Drugs Tested

The amounts of 1 mg/mL and 100 mg/mL are obtained by dilution of a solution of acetylsalicylate (Aspegic, Sanofi-synthelabo, France). Aspirin dilutions were prepared as follows: 1 g of pure, finely powdered aspirin was suspended in 99 mL of alcohol (70°). After being vigorously shaken, 1 mL of this dilution was then mixed with 99 mL of distilled water and vigorously shaken (dilution 1). The last process was repeated until obtaining desired dilutions: 4 times (dilution 5), 8 times (dilution 9), and 14 times (dilution 15). Sterilized water (placebo) or aspirin were subcutaneously administered at a final volume of 1 mL/kg mouse weight. The groups were treated with placebo or aspirin in 100 mg/kg, 1 mg/kg or dilutions 5, 9, or 15 (*n* = 9–11 mice/group).

#### 3.1.5. Distribution of Groups

COX 1 −/− or COX 2 −/− Knockout mice were distributed in 6 groups (*n* = 12/group), respectively.

Group 1: placebo (sterilized water).
Group 2: aspirin 100 mg/kg.
Group 3: aspirin 1 mg/kg.
Group 4: aspirin dilution 5.
Group 5: aspirin dilution 9.
Group 6: aspirin dilution 15.

#### 3.1.6. Statistical Analysis

Data are expressed as mean ± SD and compared using one-way analysis of variance (ANOVA) followed by Dunnett's multiple comparison test. A value of *P* < 0.05 was considered as significant. Statistical calculations were performed using GraphPad Prism version 4.00 for Windows.

### 3.2. Results (Tables 1, 2, and 3)

The IHT model is especially sensitive to the effect of high doses of ASA. However, mice without COX 1 did not react to the higher doses of ASA. The highest dilution (Dil 15) of ASA significantly shortened IHT in COX 1-deficient mice, confirming that its strong prothrombotic effect is not mediated by COX 1. No significant changes in IHT were observed after ASA in COX 2-deficient mice. 

NE and DE with placebo in COX 1-deficient mice were clearly decreased when compared to COX 2-deficient mice, highlighting the importance of COX 1 generated TXA_2_ in the platelets. The highest dose of aspirin produced a decreased NE in COX 1-deficient mice.

## 4. Discussion

Administered at high dose (100 mg/kg), aspirin showed potent antithrombotic effects. This benefit action of aspirin is associated with risk of hemorrhagic side effects.

When administered at 1 mg/kg, aspirin prevents thromboembolic complication, but in less important manner, compared with 100 mg/kg. This moderated antithrombotic effect is not associated with an increased hemorrhagic risk.

The presence of ultra-low doses of aspirin, which is equivalent to high dilution, increased the thromboembolic complications without effect on hemorrhage. These residual concentrations of aspirin could explain the advent of thromboembolic complications after aspirin discontinuation. This phenomenon required 8 to 12 days to express in clinical studies and would explain the appearance of the secondary side effects observed several days after the treatment discontinuation. 

These repeated phenomena could induce complications which are not related to the administered substances.

In conclusion, aspirin at strong dose (100 mg/kg) prevents thromboembolic complications in the rat with an important hemorrhagic risk. With lower doses (1 mg/kg) aspirin still preserves a light antithrombotic activity but without increasing the hemorrhagic risk.

Ultra-low doses of aspirin induce a risk of thromboembolic complications which are not associated with an action on the hemorrhage.

## Figures and Tables

**Table 1:** Effects of aspirin on the platelet activity in normal mice.

ASA	Placebo	100 mg/kg	1 mg/kg	Dil 5	Dil 9	Dil 15
IHT (sec)	113.9 ± 24.64	363.3 ± 93.3*	110.5 ± 97.3	119.2 ± 36.7	138.8 ± 24.1	103.6 ± 78.8
NE	4.8 ± 1.5	1.1 ± 0.7*	3.0 ± 1.2*	6.4 ± 1.9	5.6 ± 1.9	9.4 ± 1.7*
DE (min)	2.1 ± 0.7	0.3 ± 0.4*	1.1 ± 0.6*	3.0 ± 0.8	2.2 ± 0.7	4.1 ± 1.5*

*P < 0.05 indicates a statistically significant difference with the placebo group.

**Table 2:** Effects of aspirin on the platelet activity in COX 2 −/− knockout mice.

ASA	Placebo	100 mg/kg	1 mg/kg	Dil 5	Dil 9	Dil 15
IHT (sec)	327.3 ± 103.8	212.2 ± 109.3	288.1 ± 98.7	300.9 ± 131.4	336.5 ± 77.7	245.5 ± 123.9
NE	9.0 ± 3.9	3.29 ± 2.36*	7.0 ± 4.5	6.50 ± 3.2	5.25 ± 3.24	8.11 ± 2.76
DE (min)	4.5 ± 1.73	1.29 ± 1.11*	3.0 ± 2.4	2.38 ± 1.51	2.25 ± 1.58	3.55 ± 1.01

*P < 0.05 indicates a statistically significant difference with the placebo group.

**Table 3:** Effects of aspirin on the platelet activity in COX 1 −/− knockout mice.

ASA	Placebo	100 mg/kg	1 mg/kg	Dil 5	Dil 9	Dil 15
IHT (sec)	255.7 ± 92.23	288.3 ± 77.0	287.8 ± 79.2	275.0 ± 107.2	229.3 ± 80.63	164.8 ± 88.9*
NE	2.45 ± 1.21	1.36 ± 1.03*	2.09 ± 1.4	2.98 ± 1.05	2.8 ± 1.14	4.6 ± 2.01*
DE (min)	1.09 ± 0.94	0.36 ± 0.67	0.73 ± 0.79	1.3 ± 0.48	1.3 ± 0.67	2.0 ± 0.82*

*P < 0.05 indicates a statistically significant difference with the placebo group.
